# Quantification of the renal sinus fat and exploration of its relationship with ectopic fat deposition in normal subjects using MRI fat fraction mapping

**DOI:** 10.3389/fendo.2023.1187781

**Published:** 2023-08-09

**Authors:** Qin-He Zhang, Li-Hua Chen, Qi An, Peng Pi, Yi-Fan Dong, Ying Zhao, Nan Wang, Xin Fang, Ren-Wang Pu, Qing-Wei Song, Liang-Jie Lin, Jing-Hong Liu, Ai-Lian Liu

**Affiliations:** ^1^ Department of Radiology, The First Affiliated Hospital of Dalian Medical University, Dalian, China; ^2^ Department of Medical Imaging, Dalian Medical University, Dalian, China; ^3^ Clinical & Technical Solutions, Philips Healthcare, Beijing, China

**Keywords:** renal sinus fat, magnetic resonance imaging, ectopic fat deposition, obesity, fat quantification

## Abstract

**Purpose:**

To determine the renal sinus fat (RSF) volume and fat fraction (FF) in normal Chinese subjects using MRI fat fraction mapping and to explore their associations with age, gender, body mass index (BMI) and ectopic fat deposition.

**Methods:**

A total of 126 subjects were included in the analysis. RSF volume and FF, visceral adipose tissue (VAT) and subcutaneous adipose tissue (SAT) area, and hepatic and pancreatic FFs were measured for each subject. The comparisons in gender were determined using two-tailed t-tests or the nonparametric Mann-Whitney U-test for normally or non-normally distributed data for continuous variables and the chi-square test for categorical variables. Comparisons of RFS volume and FF between right and left kidneys were determined using paired sample t-tests. Multivariable logistic models were performed to confirm whether RSF differences between men and women are independent of VAT or SAT area. When parameters were normally distributed, the Pearson correlation coefficient was used; otherwise, the Spearman correlation coefficient was applied.

**Results:**

The RSF volumes (cm^3^) of both kidneys in men (26.86 ± 8.81 for right and 31.62 ± 10.32 for left kidneys) were significantly bigger than those of women (21.47 ± 6.90 for right and 26.03 ± 8.55 for left kidneys) (P < 0.05). The RSF FFs (%) of both kidneys in men (28.33 ± 6.73 for right and 31.21 ± 6.29 for left kidneys) were significantly higher than those of the women (23.82 ± 7.74 for right and 27.92 ± 8.15 for left kidneys) (P < 0.05). The RSF differences between men and women are independent of SAT area and dependent of VAT area (except for right RSF volume). In addition, the RSF volumes and FFs in both kidneys in the overall subjects show significant correlations with age, BMI, VAT area, hepatic fat fraction and pancreatic fat fraction (P < 0.05). However, the patterns of these correlations varied by gender. The RSF volume and FF of left kidney were significantly larger than those of the right kidney (P < 0.05).

**Conclusion:**

The association between renal sinus fat and ectopic fat deposition explored in this study may help establish a consensus on the normal values of RSF volume and FF for the Chinese population. This will facilitate the identification of clinicopathological changes and aid in the investigation of whether RSF volume and FF can serve as early biomarkers for metabolic diseases and renal dysfunction in future studies.

## 1 Introduction

The renal sinus (RS) is a space that forms the medial border of the kidney and is surrounded by the renal parenchyma laterally. The upper part of renal arteries, veins, lymphatic vessels, nerves, renal pelvis, and major and minor calices are all located within the RS. As a kind of ectopic fat (e.g., liver, pancreas, skeletal muscle, and/or heart fat), renal sinus fat (RSF) refers to the variable amount of ectopic perivascular fat that distributed surrounds the structures within the RS (1), and is a compartment of visceral fat. The accumulation of RSF holds significant importance as it can potentially lead to compression of the renal veins and arteries that pass through this adipose tissue (2).

RSF may have effects on hypertension, cardiovascular risk and renal dysfunction (3–5). Recently, there is increased interest on the study of association between RSF and type 2 diabetes mellitus (T2DM). Larger RSF volume has been reported to be associated with lower glomerular filtration rate (GFR) and increased renal vascular resistance in T2DM patients (6). Furthermore, RSF volume was positively associated with several metabolic risk factors including HbA1c level and urinary albumin-to-creatinine ratio in T2DM patients (7).

Because conventional anthropometric measures of adiposity fail to capture the organ-specific fat deposition, advanced imaging techniques are indispensable for exploring the plausible implications of RSF accumulation on human health. Volumetric analysis of RSF by computed tomography (CT) and magnetic resonance imaging (MRI) and assessment of its association with metabolic and cardiovascular risks have been previously reported (6–10).

However, compared with RSF volume, little is known about the quality of RSF, which can be associated with adipose tissue dysfunction (5). An MRI iterative decomposition of water and fat with echo asymmetry at least-square estimation-iron quantification (IDEAL-IQ) method enables accurate measurement of fat content, with a low flip angle for suppressing the T1 effects and multi-echo acquisition for water-fat separation and correction of the T2* effects. It is currently considered the best non-invasive technique for assessing fat fraction (FF) and fat volume (11).

The associations of pancreatic volume, pancreatic fat, and hepatic fat with age, gender, and ectopic fat have been explored in normal subjects in previous studies (12–18). However, also as a kind of ectopic fat, RSF and its physiological changes in normal subjects are still unclear. To the best of our knowledge, to date, there was lack of studies about investigating normal quantitative metrics of the RSF volume and especially RSF FF as well as their correlations with biometric parameters (e.g., age and gender) and other ectopic adipose tissues (e.g., hepatic fat and pancreatic fat).

In this study, we hypothesized that RSF was associated with age, gender, body mass index (BMI) and ectopic fat deposition. Therefore, the purpose of this study was to determine RSF volume and FF in normal Chinese subjects using MRI fat fraction mapping and to assess the associations of RSF with age, gender, BMI and ectopic fat deposition.

## 2 Materials and methods

### 2.1 Study population

This was a retrospective study of clinically suspicious cases of abdominal disease between January 2017 and August 2020. All patients underwent upper-middle abdominal MRI examination (including IDEAL-IQ imaging). Similar to the previous studies (10, 19, 20), participants who met at least one of the following criteria were excluded (1): age < 18 years (2); a history of heavy drinking (alcohol consumption ≥ 30 g per week in men or ≥ 20 g per week in women in the last 10 years) (3); evidence of cirrhosis, malignant liver tumor, large benign liver tumor, liver post-hepatectomy, decompensated liver diseases; evidence of other liver diseases, including viral hepatitis, autoimmune liver diseases, drug-induced liver injury, etc. (4); evidence of pancreas diseases (5); intrahepatic bile or pancreatic duct dilation (6); evidence of ascites, mesenteric injuries, huge abdominal mass, abdominal wall edema, post-ostomy (7); radiotherapy, chemotherapy, immunosuppressive therapy, antiviral therapy, and endocrine therapy (8); pregnancy (9); hypertension and/or T2DM (10); a history of renal surgery, hydronephrosis, renal sinus mass, renal dysfunction or renal malformation. Finally, a total of 126 subjects (46 men and 80 women) were included in the analysis. The Ethical Committee approved the study.

### 2.2 MRI examinations

In this study, the MRI scanner (GE Medical Systems, Inc., Waukesha, WI, USA) with an eight-channel phased-array body coil was used. The patients fasted for 4-6 hours and were trained to exhale and hold their breath for more than 20 seconds before scanning. The subjects were placed in the supine position during examination. A three-plane localization imaging gradient-echo sequence was performed at the beginning of acquisition. IDEAL-IQ was acquired and scanning parameters were shown in **Table 1**.

**Table 1:** IDEAL-IQ scanning parameters.

Field strength (T)	TE (ms)	TR (ms)	FOV (cm^2^)	Matrix	NEX	Slice thickness (mm)
1.5	13.4	4.8	36 × 36	256 × 160	1	10
3.0	6.9	3.0	36 × 36	256 × 160	1	10

TE, echo time; TR, repetition time; FOV, field of view; NEX, number of excitations.

### 2.3 Data measurements

#### 2.3.1 Measurement of renal sinus fat

**Figure 1:**
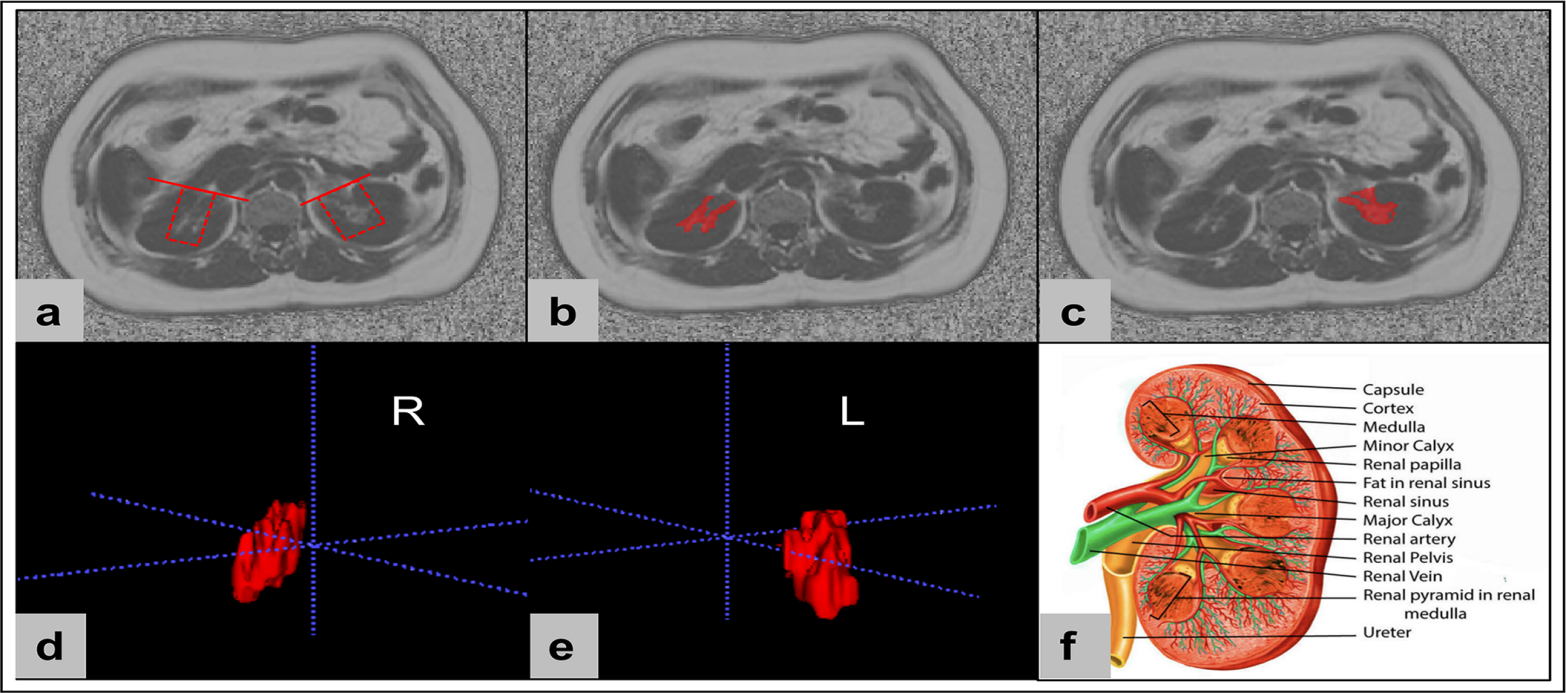
The renal sinus fat was manually conducted using the open-source software ITK-SNAP (v.3.6.0, http://www.itksnap.org/). Renal sinus fat of both kidneys was identified on MRI fat fraction maps by a straight-line tangent to the margins of parenchyma beside the renal hilum in axial slices **(A)**. Then the bilateral renal sinus adipose tissues were manually segment with structures of renal lymphatics, veins, and the ureters removed **(B, C)**. Finally, the renal sinus fat volume and FF of right and left kidneys were automatically calculated based on the 3D segmentations **(D, E)**. Sketch of the renal anatomy **(F)** (Source: [21] distributed under the terms of the Creative Commons Attribution 4.0 International License (https://reativecommons.org/licenses/by/4.0/, accessed on the 1 March 2023) © 2023, StatPearls Publishing LLC).

RSF was defined by an ectopic perivascular fat depot around renal hilum, which is in close contact with renal vasculature, lymphatic vessel, renal pelvis and calyces (10). All slices of upper-middle abdominal MRI fat fraction map were chosen for RSF analysis (about 20 – 24 slices, slice thickness 10 mm) using the open-source software ITK-SNAP (v.3.6.0, http://www.itksnap.org/), and RSF was manually segmented. Based on the sketch of the renal anatomy (21), RSF of both kidneys was identified on MRI FF map by a straight-line tangent to the margins of parenchyma beside the renal hilum in axial slices. Then the bilateral renal sinus adipose tissue manually segmented with structures of renal lymphatics, veins, and the ureters removed. Since adipose tissue exhibits high signal intensity on the FF map, the labeling of RSF started from the upper pole of the kidney with the high signal tissues in the renal sinus area labelled, and continued downward until reaching the lower pole of the kidney (**Figure 1**). Finally, the RSF volume and FF of right and left kidneys were automatically calculated using homemade software based on MATLAB (MATLAB R2018a).

#### 2.3.2 Measurement of visceral adipose tissue and subcutaneous adipose tissue

Area (cm^2^) of VAT and SAT was semi-automatically measured on the axial FF images by Image J (National Institutes of Health, USA), as previously described (11). The abdominal fat was determined at the L1-L2 level and did not include intestinal loops.

#### 2.3.3 Measurement of hepatic FF

On the post-processing platform (Intellispace portal v9.0, ISP v9.0, Philips Healthcare, Best, the Netherland), the software algorithm (multimodality tumor tracking, MMTT) could recognize the 3D margins of the liver on FF maps, and the whole liver was then semi-automatically traced with necessary manual corrections. The main portal vein, inferior vena cava, and the gallbladder were manually removed. After liver segmentation, the whole hepatic FF was automatically calculated (11).

#### 2.3.4 Measurement of pancreatic FF

The whole pancreatic FF was calculated using the same method as for liver avoiding extra-pancreatic adipose tissue and vessel (11).

### 2.4 Inter- and intra-observer variability

The intra- and inter-observer variability were determined by repeated analysis of 30 random selected patients more than 4 weeks apart by the same observer and by the renal sinus fat measurements of the same patient by a second independent observer. Two radiologists were blinded to the grouping.

### 2.5 Statistical analysis

All data were analyzed using GraphPad Prism (Version 8.4.0, GraphPad software, LLC). The Kolmogorov-Smirnov test was used to test the normality of the variables. Normally distributed data were expressed as means ± standard deviations, and non-normally distributed data were expressed as medians and ranges (25th, 75th percentiles). The intraclass correlation coefficient (ICC) was used to check the consistency of the two observers: ICC < 0.4 indicated poor consistency; 0.4 ≤ ICC ≤ 0.75 indicated moderate consistency; ICC > 0.75 indicated good consistency. The comparisons of RFS volume and FF, and clinical and demographic characteristics in gender were determined using the independent-sample t tests for normally distributed continuous variables, and using the non-parametric Mann-Whitney U-test for non-normally distributed continuous variables. Multiple linear regression was performed to confirm whether RSF volume and FF differences between men and women are independent of VAT or SAT area. The comparisons of RFS volume and FF between right and left were determined using paired sample t-tests. When parameters were normally distributed, the Pearson correlation coefficient was used; otherwise, the Spearman correlation coefficient was applied. Correlation coefficients were interpreted as follows: weak, 0 - 0.4; moderate, 0.4 - 0.7; and strong, 0.7 - 1.0. In addition, we performed sex-stratified analyses. A two-tailed P value < 0.05 was considered to be statistically significant. There was no multiplicity adjustment of P-values.

## 3 Results

### 3.1 Study sample characteristics

A total of 126 patients with a median age of 56 years and a mean BMI of 23.64 kg/m^2^ were finally included in the study. Clinical and demographic characteristics are summarized in **Table 2**.

**Table 2:** Clinical characteristics of the study subjects.

Characteristics	Overall subjects	Men	Women	P-value*
N	126	46	80	
Age, years	56 (46, 62.25)	54 (42, 63)	56 (48.25, 61)	0.616
Weight, kg	65.00 (59.00, 72.50)	73.00 (67.00, 79.00)	60.00 (56.5, 67.00)	< 0.001
Height, m	1.65 (1.62, 1.73)	1.75 (1.73, 1.76)	1.63 (1.60, 1.65)	< 0.001
BMI, kg/m^2^	23.64 ± 3.05	24.09 ± 2.70	23.41 ± 3.22	0.227
RSF FF of left kidney, %	29.09 ± 7.68	31.21 ± 6.29	27.92 ± 8.15	0.020
RSF volume of left kidney, cm^3^	28.07 ± 9.58	31.62 ± 10.32	26.03 ± 8.55	0.003
RSF FF of right kidney, %	25.41 ± 7.68	28.33 ± 6.73	23.82 ± 7.74	0.001
RSF volume of right kidney, cm^3^	23.38 ± 8.06	26.86 ± 8.81	21.47 ± 6.90	0.001
SAT area, cm^2^	120.23 (87.71, 153.72)	94.48 (72.84, 121.30)	131.04 (109.42, 173.81)	< 0.001
VAT area, cm^2^	122.65 (66.29, 152.97)	143.57 (114.21, 185.70)	106.26 (54.35, 135.28)	0.001
Hepatic fat fraction, %	3.30 (2.58,4.65)	3.30 (2.60, 4.90)	3.25 (2.50, 4.60)	0.626
Pancreatic fat fraction, %	5.55 (3.80, 8.03)	6.10 (4.18, 8.18)	5.35 (3.58, 7.95)	0.306
Systolic BP, mmHg	120 (110, 120)	120 (115,120)	120 (110, 120)	0.058
Diastolic BP, mmHg	75 (70, 80)	78 (70, 80)	70 (70, 80)	0.173
FPG, mmol/L	4.92 (4.68, 5.32)	4.91 (4.71, 5.29)	4.93 (4.68, 5.32)	0.893
TG, mmol/L	1.07 (0.82, 1.56)	1.06 (0.82, 1.58)	1.08 (0.83, 1.55)	0.952
TC, mmol/L	4.82 ± 1.09	4.59 ± 0.97	4.95 ± 1.14	0.072
HDL-C, mmol/L	1.30 (1.02, 1.47)	1.10 (0.93, 1.37)	1.39 (1.17, 1.66)	< 0.001
LDL-C, mmol/L	2.66 ± 0.83	2.61 ± 0.76	2.70 ± 0.88	0.551

*P-value shows comparison of men and women; N-the number of participants in each group.

The differences in age, height, weight, SAT area, VAT area, hepatic fat fraction, pancreatic fat fraction, BP, FPG, TG and HDL-C between men and women were analyzed by the non-parametric Mann-Whitney U-test; the differences in BMI, right RSF FF and volume, left RSF FF and volume, TC, LDL-C between men and women were analyzed by the independent-sample t tests.

### 3.2 Consistency analysis

The data consistency is shown in **Table 3**. The ICC values were all higher than 0.9, which suggested good inter-observer and intra-observer agreement.

**Table 3:** Two-observer measurement consistency.

	Radiologist A1	Radiologist A2	ICC 1^*^	Radiologist B	ICC 2^*^
RSF FF of left kidney, %	30.24 ± 7.40	32.54 ± 7.30	0.950	31.67 ± 7.33	0.958
RSF volume of left kidney, cm^3^	29.73 (25.63, 34.29)	26.93 ± 7.54	0.903	27.61 ± 7.35	0.908
RSF FF of right kidney, %	25.02 ± 6.29	26.32 ± 6.43	0.979	26.38 ± 6.40	0.942
RSF volume of right kidney, cm^3^	23.46 (18.77, 28.87)	21.86 ± 6.54	0.959	22.55 ± 6.90	0.951
SAT area, cm^2^	128.96 (84.90, 165.57)	127.19 (90.27, 168.10)	0.990	135.72 (82.76, 162.94)	0.984
VAT area, cm^2^	111.84 (76.10, 148.70)	112.39 (82.74, 154.53)	0.992	112.36 (84.59, 152.43)	0.992
Hepatic fat fraction, %	3.10 (2.60, 4.30)	3.15 (2.50, 4.40)	0.996	3.35 (2.60, 4.30)	0.998
Pancreatic fat fraction, %	5.35 (4.00, 7.80)	5.65 (4.30, 8.00)	0.999	5.65 (4.30, 8.30)	0.999

^*^ICC 1 shows ICC value of Intra-observer and ICC 2 shows ICC value of Inter-observer.

### 3.3 Correlations between the renal sinus fat and gender

**Figure 2:**
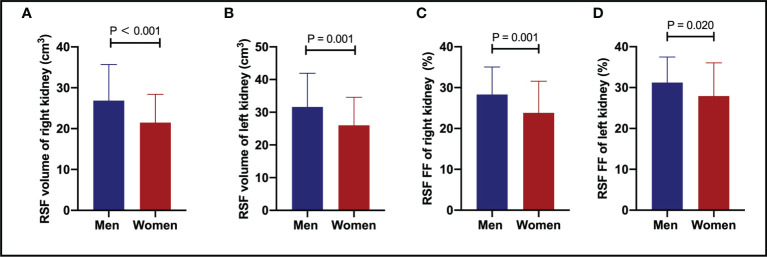
The comparisons of RSF volumes of right **(A)** and left **(B)** kidneys, and RSF FFs of right **(C)** and left **(D)** kidneys between genders. Both the RSF volume and FF of both kidneys in men were significantly larger than those in women (P < 0.05). RSF, renal sinus fat; FF, fat fraction.

It was found that the RSF volumes (cm^3^) in men were 26.86 ± 8.81 for right and 31.62 ± 10.32 for left kidneys. The RSF volumes in women (cm^3^) were 21.47 ± 6.90 for right and 26.03 ± 8.55 for left kidneys. The RSF volumes of both kidneys in men were significantly higher than those in women (P < 0.05) (**Table 2**; **Figure 2**).

In addition, it was also found that the RSF FFs (%) in men were 28.33 ± 6.73 for right and 31.21 ± 6.29 for left kidneys. The RSF FFs in women (%) were 23.82 ± 7.74 for right and 27.92 ± 8.15 for left kidneys. The RSF FFs of both kidneys in men were significantly higher than those in women (P < 0.05) (**Table 2**; **Figure 2**).

Furthermore, results of linear regression analyses for RSF volume and FF between men and women are shown in **Table 4**. The results showed that all RSF differences between men and women are independent of SAT area, and there were more RSF volume and FF in men (All β > 0). Yet, after adjusting for VAT area, only the right RSF volume showed significant difference between men and women.

**Table 4:** Multiple linear regression for RSF volume and FF of left and right kidney in gender.

Models	Gender			Gender + VAT area			Gender + SAT area		
	β*	95%CI	P-value	β*	95%CI	P-value	β*	95%CI	P-value
Right RSF volume	5.384	2.583 – 8.186	< 0.001	3.021	0.385 – 5.656	0.025	6.867	3.895 – 9.838	< 0.001
Right RSF FF	4.507	1.800 - 7.214	0.001	—	—	—	6.364	3.545 – 9.183	< 0.001
Left RSF volume	5.592	2.211 – 8.972	0.001	—	—	—	7.794	4.257 – 11.330	< 0.001
Left RSF FF	3.293	0.537 – 6.049	0.020	—	—	—	4.902	1.996 – 7.809	0.001

CI, confidence interval; β > 0 indicated that there were more RSF volume or FF in men.

*β is only representative for gender.

### 3.4 Correlations between the renal sinus fat and age

**Figure 3:**
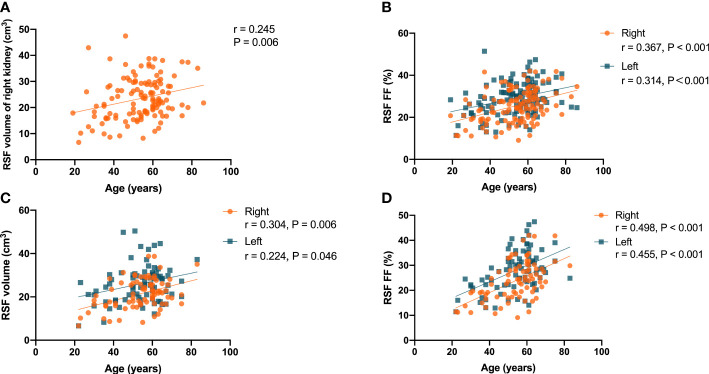
Correlation of the RSF volume in right kidney with the age in all subjects **(A)**; correlations the RSF FF in both kidneys with age in all subjects **(B)**; correlations of the RSF volume **(C)** and RSF FF **(D)** in both kidneys with the age in women subjects.

It was found that there was a significant correlation (P = 0.006, r = 0.245) in the overall subjects between the RSF volume of right kidney and the age (**Figure 3A**), but not for RSF volume of the left kidney (P = 0.075, r = 0.159). In addition, there were significant correlations (P < 0.001, r = 0.367 for right kidney, and P<0.001, r = 0.314 for left kidney) between the RSF FFs in both kidneys and the age in the overall subjects (**Figure 3B**).

In women, there were correlations (P = 0.006, r = 0.304 for right kidney, and P = 0.046, r = 0.224 for left kidney) between the RSF volumes in both kidneys and the age (**Figure 3C**). In addition, there were correlations (P < 0.001, r = 0.498 for right kidney, and P < 0.001, r = 0.455 for left kidney) between the RSF FFs in both kidneys and the age (**Figure 3D**).

However, there was no significant correlation of RSF volumes or FFs in both kidneys with the age in men.

### 3.5 Correlations between the renal sinus fat and BMI

**Figure 4:**
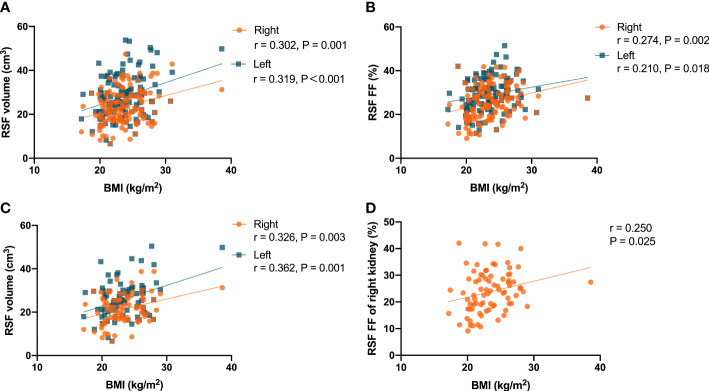
Correlations between the RSF volumes in both kidneys and BMI in the all subjects **(A)**; Correlations between the RSF FFs in both kidneys and BMI in the all subjects **(B)**; Correlations between the RSF volumes in both kidneys and BMI in women subjects **(C)**; Correlation between the RSF FF in right kidney and BMI in women subjects **(D)**.

It was found that there were correlations (P = 0.001, r = 0.302 for right kidney, and P<0.001, r = 0.319 for left kidney) of the RSF volumes in both kidneys with BMI in the overall subjects (**Figure 4A**). In addition, it was also found that there were correlations (P = 0.002, r = 0.274 for right kidney, and P = 0.018, r = 0.210 for left kidney) of the RSF FFs in both kidneys with BMI in the overall subjects (**Figure 4B**).

In women, it was found that there were correlations (P = 0.003, r = 0.326 for right kidney, and P = 0.001, r = 0.362 for left kidney) between the RSF volumes in both kidneys and BMI (**Figure 4C**). In addition, it was also found that there was a correlation (P = 0.025, r = 0.250) between the RSF FF in right kidney and BMI, but not for the RSF FF in left kidney (P = 0.138, r = 0.167) (**Figure 4D**).

However, in men, it was found that there was no correlation (P = 0.120, r = 0.232 for right kidney, and P = 0.160, r = 0.213 for left kidney) between the RSF volumes in both kidneys and BMI. In addition, there was no correlation (P = 0.073, r = 0.267 for right kidney, and P = 0.079, r = 0.262 for left kidney) between the RSF FFs in both kidneys and BMI.

### 3.6 Correlations between the renal sinus fat and SAT

It was found that there was no significant correlation in the overall subjects between the RSF volume and FF in both kidneys and the SAT area.

**Figure 5:**
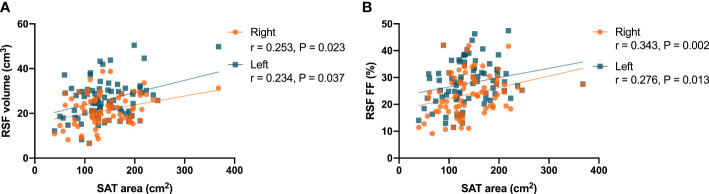
Correlations between the RSF volumes in both kidneys and the SAT area **(A)** in women; Correlations between the RSF FFs in both kidneys and the SAT area **(B)** in women.

In women, it was found that there were correlations (P = 0.023, r = 0.253 for right kidney, and P = 0.037, r = 0.234 for left kidney) between the RSF volumes in both kidneys and the SAT area (**Figure 5A**). In addition, it was found that there were correlations (P = 0.002, r = 0.343 for right kidney, and P = 0.013, r = 0.276 for left kidney) between the RSF FFs in both kidneys and the SAT area (**Figure 5B**).

However, in men, it was found that there was no significant correlation between the RSF volumes and FFs in both kidneys and the SAT area.

### 3.7 Correlations between the renal sinus fat and VAT

**Figure 6:**
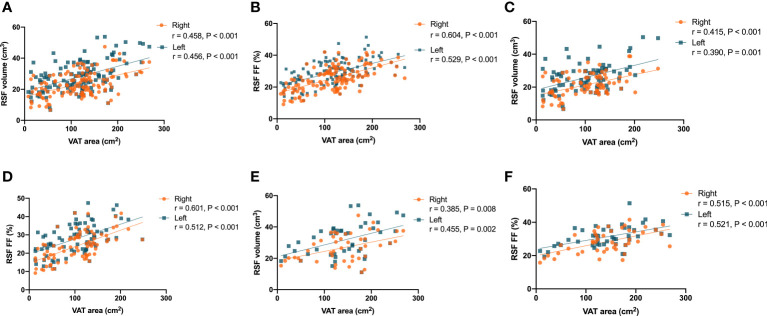
Correlations between the RSF volumes in both kidneys and the VAT area in the overall subjects **(A)**; Correlations between the RSF FFs in both kidneys and the VAT area in the overall subjects **(B)**; Correlations between the RSF volumes in both kidneys and the VAT area in women **(C)**; Correlations between the RSF FFs in both kidneys and the VAT area in women **(D)**; Correlations between the RSF volumes in both kidneys and the VAT area in men **(E)**; Correlations between the RSF FFs in both kidneys and the VAT area in men **(F)**.

It was found that there were correlations (P < 0.001, r = 0.458 for right kidney, and P < 0.001, r = 0.456 for left kidney) in the overall subjects between the RSF volumes in both kidneys and the VAT area (**Figure 6A**). In addition, it was also found that there were correlations (P < 0.001, r = 0.604 for right kidney, and P < 0.001, r = 0.529 for left kidney) in the overall subjects between the RSF FFs in both kidneys and the VAT area (**Figure 6B**).

The correlations between RSF volumes and FFs in both kidneys of different genders and the VAT area were similar to that observed in the total study population. In women, it was found that there were correlations (P < 0.001, r = 0.415 for right kidney, and P = 0.001, r = 0.390 for left kidney) between the RSF volumes in both kidneys and the VAT area (**Figure 6C**). In addition, it was also found that there were correlations (P < 0.001, r = 0.601 for right kidney, and P < 0.001, r = 0.512 for left kidney) between the RSF FFs in both kidneys and the VAT area (**Figure 6D**).

In men, it was found that there were correlations (P = 0.008, r = 0.385 for right kidney, and P = 0.002, r = 0.455 for left kidney) between the RSF volumes in both kidneys and the VAT area (**Figure 6E**). In addition, it was also found that there were correlations (P < 0.001, r = 0.515 for right kidney, and P < 0.001, r = 0.521 for left kidney) between the RSF FFs in both kidneys and the VAT area (**Figure 6F**).

### 3.8 Correlations between the renal sinus fat and hepatic fat fraction

**Figure 7:**
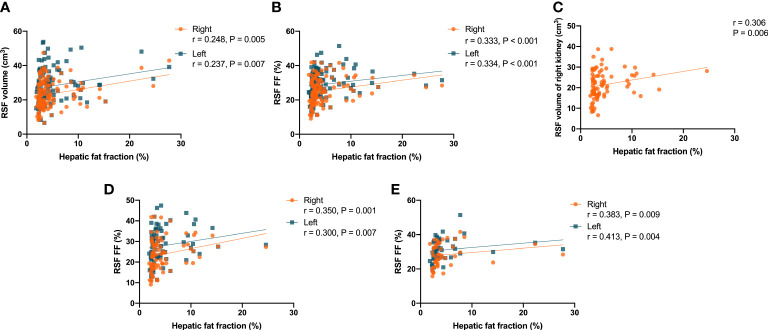
Correlations between the RSF volumes in both kidneys and the hepatic fat fraction in the overall subjects **(A)**; Correlations between the RSF FFs in both kidneys and the hepatic fat fraction in the overall subjects **(B)**; Correlation between the RSF volume in right kidney and the hepatic fat fraction in women **(C)**; Correlations between the RSF FFs in both kidneys and the hepatic fat fraction in women **(D)**; Correlations between the RSF FFs in both kidneys and the hepatic fat fraction in men **(E)**.

It was found that there were correlations (P = 0.005, r = 0.248 for right kidney, and P = 0.007, r = 0.237 for left kidney) in the overall subjects between the RSF volumes in both kidneys and the hepatic fat fraction (**Figure 7A**). In addition, it was also found that there were correlations (P < 0.001, r = 0.333 for right kidney, and P < 0.001, r = 0.334 for left kidney) in the overall subjects between the RSF FFs in both kidneys and the hepatic fat fraction (**Figure 7B**).

In women, it was found that there was a correlation (P = 0.006, r = 0.306) between the RSF volume in right kidney and the hepatic fat fraction, but not for the RSF volume in left kidney (P = 0.070, r = 0.203) (**Figure 7C**). In addition, it was also found that there were correlations (P = 0.001, r = 0.350 for right kidney, and P = 0.007, r = 0.300 for left kidney) between the RSF FFs in both kidneys and the hepatic fat fraction (**Figure 7D**).

However, in men, it was found that there was no correlation between the RSF volumes in both kidneys and the hepatic fat fraction. Yet, it was found that there were correlations (P = 0.009, r = 0.383 for right kidney, and P = 0.004, r = 0.413 for left kidney) between the RSF FFs in both kidneys and the hepatic fat fraction (**Figure 7E**).

### 3.9 Correlations between the renal sinus fat and pancreatic fat fraction

**Figure 8:**
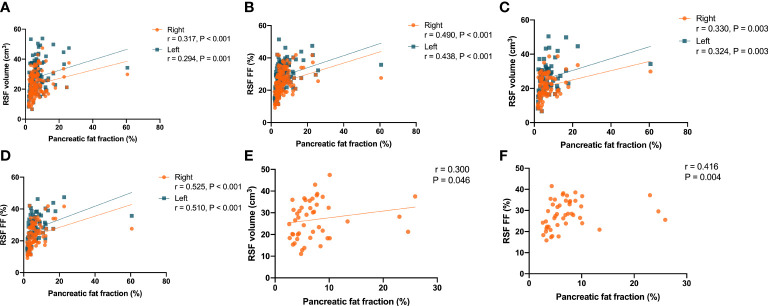
Correlations between the RSF volumes in both kidneys and the pancreatic fat fraction in the overall subjects **(A)**. Correlations between the RSF FFs in both kidneys and the pancreatic fat fraction in the overall subjects **(B)**. Correlations between the RSF volumes in both kidneys and the pancreatic fat fraction in women **(C)**. Correlations between the RSF FFs in both kidneys and the pancreatic fat fraction in women **(D)**. Correlation between the RSF volume in right kidney and the pancreatic fat fraction in men **(E)**. Correlation between the RSF FF in right kidney and the pancreatic fat fraction in men **(F)**.

It was found that there were correlations (P < 0.001, r = 0.317 for right kidney, and P = 0.001, r = 0.294 for left kidney) between the RSF volumes in both kidneys and the pancreatic fat fraction in the overall subjects (**Figure 8A**). In addition, it was also found that there were correlations (P < 0.001, r = 0.490 for right kidney, and P < 0.001, r = 0.438 for left kidney) in the overall subjects between the RSF FFs in both kidneys and the pancreatic fat fraction (**Figure 8B**).

The correlations between RSF volumes and FFs in both kidneys and the VAT area in women were similar to that observed in the overall subjects. it was found that there were correlations (P = 0.003, r = 0.330 for right kidney, and P = 0.003, r = 0.324 for left kidney) between the RSF volumes in both kidneys and the pancreatic fat fraction (**Figure 8C**). In addition, it was also found that there were correlations (P < 0.001, r = 0.525 for right kidney, and P < 0.001, r = 0.510 for left kidney) between the RSF FFs in both kidneys and the pancreatic fat fraction (**Figure 8D**).

However, in men, it was found that there was only a correlation (P = 0.046, r = 0.300) between the RSF volume in right kidney and the pancreatic fat fraction, but not for the RSF volume in left kidney (P = 0.141, r = 0.221) (**Figure 8E**). In addition, it was found that there was a correlation (P = 0.004, r = 0.416) between the RSF FF in right kidney and the pancreatic fat fraction, but not for the RSF FF in left kidney (P = 0.141, r = 0.221) (**Figure 8F**).

### 3.10 Difference between the renal sinus fat of the left and right kidney

**Figure 9:**
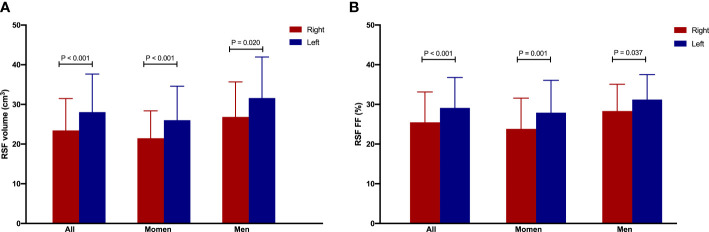
The RSF volume **(A)** and FF **(B)** of left kidney were significantly larger than those of the right kidney in overall subjects or in women or men group separately.

It was found that the RSF volumes (cm^3^) were 23.38 ± 8.06 for right and 28.07 ± 9.58 for left kidneys, and RSF FFs (%) were 25.41 ± 7.68 for right and 29.09 ± 7.68 for left kidneys in overall subjects. In addition, we found that the RSF volumes (cm^3^) were 21.47 ± 6.90 for right and 26.03 ± 8.55 for left kidneys, and RSF FFs (%) were 23.82 ± 7.74 for right and 27.92 ± 8.15 for left kidneys in women; and the RSF volumes (cm^3^) were 26.86 ± 8.81 for right and 31.62 ± 10.32 for left kidneys, and RSF FFs (%) were 28.23 ± 6.73 for right and 31.21 ± 6.29 for left kidneys in men. The RSF volume and FF of left kidney were significantly larger than those of the right kidney in overall subjects or in different gender groups separately (all P < 0.05, **Figure 9**).

## 4 Discussion

This study yielded several outcomes. First, the RSF volume and FF values of both kidneys in men were significantly higher than those in women, but the differences were not independent of VAT area (except for right RSF volume). Second, there were significant correlations of the RSF volume and FF values in both kidneys with the age, BMI, VAT area, hepatic fat fraction and pancreatic fat fraction in the overall subjects, but not with SAT area. Yet, patterns of these correlations were varied by genders. Finally, we also found that the RSF volume and FF values of left kidney were significantly higher than those of the right kidney in the overall, regardless of gender.

Adipose tissue located in different body compartments may play distinct roles and contribute to comorbidities through unique pathophysiological mechanisms (22). The renal calyces, renal pelvis, and blood vessels were surrounded by varied amounts of adipose tissue in the renal sinus. In the metabolically benign condition, RSF was beneficial to glomerular cells, and it may promote regeneration and anti-fibrosis effects, and reduce the release of pro-inflammatory factors by endothelial cells and podocytes. However, in a metabolically malignant condition, first, excessive fat accumulation in the RS would result in increased intra-abdominal pressure and compression of structures within the RS, which increases renal hydrostatic pressure and activates the renin–angiotensin–aldosterone system (RAAS). Activation of the RAAS promotes hypertension, insulin resistance, atherosclerosis, and other adverse physiological effects related to obesity (23, 24). In addition, as an ectopic perivascular fat, RSF has the paracrine effect of secreting inflammatory cytokines and vasoconstrictive factors, which could lead to local inflammation, oxidative stress, lipotoxicity and fibrosis (3). Findings from the Framingham Heart Study has revealed that RSF was related to multiple cardiometabolic risk factors (20). It was found that RSF, as a perivascular fat depot in close contact with the adventitia of large, medium, and small arteries, has unique features that differ from other fat depots, and undergoes changes early in the development of metabolic diseases (10). Krievina et al. (4) found that the RSF was directly related to the early renal injury markers Skim-1 and FGF-21. Lee et al. (5) revealed that both metabolic syndrome and obesity were associated with lower RSF fat attenuation index. A randomized controlled trial demonstrated that increased RSF was directly associated with hypertension (25). Recently, it was also found that the accumulation of RSF seems to be involved in the pathogenesis of hypertension in obesity, and following bariatric surgery, loss of RSF was associated with remission from hypertension (1). Therefore, it is important to quantitatively assess RSF in normal participants and explore its relationship with ectopic fat deposition for early intervention in metabolism-related diseases and renal dysfunction.

Our finding confirmed the conclusions from previous cross-sectional studies (7, 19, 26), which all showed that RSF volume was positively correlated with age in normal subjects. Similarly, in present study, the RSF volume of right kidney and the RSF FFs of both kidneys in the overall subjects, as well as the RSF volume and FF values of both kidneys in women were positively correlated with age. Yet, we did not find any correlation between the RSF volume or FF value of either kidney in men and the age. It could be due to the relatively small sample size, and the race and gender heterogeneity (27–30). These observations may allow clinicians to estimate the age-related RSF volume and FF changes better and help decision making.

Nevertheless, we addressed the associations of and RSF volume and FF values with VAT area, SAT area, hepatic fat fraction, and pancreatic fat fraction. Previous studies have shown that RSF deposition was related to other deleterious fat depots, such as VAT and hepatic fat (8, 10, 20, 31). In our study, we further extended these findings and found that there were correlations of the RSF volumes and FFs in both kidneys with VAT area, hepatic fat fraction and pancreatic fat fraction in the overall subjects. VAT has the strong lipolytic activity (32), leading to hepatic and pancreatic steatosis (33, 34), and RSF is also considerably affected by VAT (35). These findings suggested that RSF may cluster with high amounts of VAT, hepatic and pancreatic fat, thus possibly contributing to their increased risk for related metabolic diseases as they grow up. However, we did not detect any association between RSF and SAT area. Similar findings can also be showed in studies on pancreatic fat infiltration and hepatic fat infiltration (15, 36). We thought that this may be attributed to the differences in metabolism between SAT and VAT, and that excess VAT is unhealthier than excess SAT (37). Contrary to our results, Yalçın et al. (9) found an association between the RSF and SAT. The different ranges of age, VAT and SAT may be the reasons for inconsistent results, and further studies are needed to more accurately explain the relationship between RSF and VAT, SAT.

We subsequently elucidated the effect of gender on RSF volume and FF. It was found that RSF volume and FF were higher in men than that in women, but the differences were not independent of VAT area (except for right RSF volume). Additionally, it is worth noting that the patterns of correlations between RSF and age, BMI as well as ectopic adipose tissue varied by gender.

At present, the underlying mechanism of the discrepancies in RSF between men and women is unclear, but may be associated with the following reasons: (1) Kidney volume: Previous results showed that renal parenchymal volume was related with body size (38, 39). The increase in renal parenchymal volume in proportion to body size during development results from nephron hypertrophy is presumably to response to the greater metabolic demand of a larger body size (40, 41). It had been found that RSF volume was associated with renal parenchyma volume (7), and previous studies have also found that both kidney volumes in men were significantly larger than those in women (6, 19, 42). In the current study, there was larger height and weight in men than those in women, which may result in larger RSF in men. (2) Different types of obesity: previous studies have indicated that body fat distribution varies by gender (43), and males exhibit a greater propensity to more VAT, whereas premenopausal females have a predilection for accumulating SAT; however, postmenopausal females are inclined to accrue greater amounts of VAT (44–48). Our findings indicated that, with BMI being equal, males have a higher VAT, whereas females exhibit a higher SAT. It is noteworthy that our multivariate analysis showed that the gender-based difference of RSF is not moderated by the variation in SAT; while, as a component of VAT, RSF differences are largely mediated by the amount of VAT. Interestingly, the difference in the right RSF volume between males and females is not influenced by VAT, and it may be an independent indicator for evaluating gender differences in obesity. (3) Sex hormones: sex hormones play a key role in the distribution pattern of adipose tissue (49). Estrogens regulate energy balance in adipose tissue, acting on both lipogenesis and lipolysis processes to affect the expansion and remodeling of adipose tissue (50). Estrogens have also been shown to enhance the storage of SAT but not VAT, and the elevated accumulation of VAT observed in postmenopausal women may be attributed to the decline of estrogen levels (46, 50, 51).

Additionally, our study showed that the RSF volume of the left kidney was significantly larger than that of the right kidney, and the result was consistent with previous studies. Cross-sectional studies revealed that there was more RSF accumulated in the left RS than right RS (4, 26). Especially, we further found that the RSF FF of left kidney was significantly higher than that of the right kidney in overall subjects. The reason may be that the asymmetric accumulation of RSF could be result of anatomical differences between the left and right renal veins (4). The right renal vein receives blood from the right kidney only, but the left renal vein receives blood from the left renal vein as well as the left gonadal and adrenal veins (52). The average left renal blood flow demonstrated a significant reduction relative to the average right renal blood flow. Additionally, there existed a substantial variation in the orientation of the calyces, given that the angles of the calyces in the right and left renal organs exhibit notable dissimilarities (53). A potential hypothesis for the notable increase in RSF accumulation in the left kidney is the presence of an innate structural phenomenon that may result in asymmetrical blood flow to the renal organs (4).

The strength of our study is that we incorporated the highly reproducible qualitative and quantitative measures of RSF by MRI fat fraction mapping and investigated the associations of RSF with age, gender, BMI and ectopic fat deposition. MRI fat fraction mapping by IDEAL-IQ is rapid, fairly accurate, non-invasive and relatively easy to perform, and it has been widely used in clinical applications. In our study, we showed that the quality as well as quantity of adipose tissue in RSF measured on MRI scans are associated with age, gender, BMI and ectopic fat deposition and to establish the RSF volume and FF population data of normal subjects in China. Especially, because the quality of adipose tissue can be reflected as FF on MRI fat fraction maps, it can be assumed that significantly higher FF values may suggest dysfunctional adipose tissue (11).

However, there were several limitations to our study. First, this retrospective study could not infer cause and effect, and a longitudinal study would be necessary to investigate the progression of RSF with age and obesity in healthy volunteers. Second, the RSF was manually segmented, which was time-consuming and subjectively dependent. In future studies, the automatic segmentation method should be explored. Third, our study sample was composed of normal Chinese subjects, so it may limit the generalization of our results to other racial population. Finally, the enrolled subjects were relatively small, and we still need to increase the sample size to provide more reliable data support for our findings. Fourth, subsequent research should explore deeper the association between RSF volume, FF and kidney diseases and metabolic disorders.

## 5 Conclusion

In this study, we demonstrated that the RSF volume and FF values of both kidneys in men were significantly higher than those of women. And the RSF volume and FF values of left kidney were significantly higher than those of the right kidney. In addition, there were significant correlations observed for the RSF volume and FF values in both kidneys with BMI, VAT area, hepatic fat fraction and pancreatic fat fraction in the overall subjects, but not with the SAT area. However, the patterns of these correlations varied by gender. These findings may help establish a consensus on the normal values of RSF volume and FF for the Chinese population. This will facilitate the identification of clinicopathological changes and aid in the investigation of whether RSF volume and FF can serve as early biomarkers for metabolic diseases and renal dysfunction in future studies.

## Data availability statement

The raw data supporting the conclusions of this article will be made available by the authors, without undue reservation.

## Ethics statement

The studies involving human participants were reviewed and approved by First Affiliated Hospital of Dalian Medical University. Written informed consent for participation was not required for this study in accordance with the national legislation and the institutional requirements. Written informed consent was not obtained from the individual(s) for the publication of any potentially identifiable images or data included in this article.

## Author contributions

In this work, XF participated in the data collection and analysis, and participated in the writing and revision of the article, actively cooperated with all aspects of the work and agreed to finally approved the version to be published. All authors had substantial contributions (1) to conception and design, acquisition of data, or analysis and interpretation of data (2), drafting the article or revising it critically for important intellectual content, and (3) final approval of the version to be published.
